# The parallel mediation effects of nutrition, physical activity on depression and sarcopenia risk among older people with diabetes

**DOI:** 10.3389/fpubh.2025.1655640

**Published:** 2025-08-11

**Authors:** Wang Miaomiao, Liu Qiao, Guo Xiaodi, Hu Xiling, Cheng Li

**Affiliations:** ^1^School of Medicine, Sias University, Zhengzhou, Henan, China; ^2^School of Nursing, Sun Yat-sen University, Guangzhou, Guangdong, China; ^3^Department of Endocrinology and Metabolism, The Third Affiliated Hospital of Sun Yat-sen University, Guangzhou, China

**Keywords:** older adult patients, diabetes, sarcopenia risk, depression, nutrition, physical activity

## Abstract

**Background:**

Depression and sarcopenia pose significant health challenges for older adults with diabetes. While previous studies have established a positive association between depression and the risk of sarcopenia, the underlying mechanisms driving this relationship remain poorly understood.

**Objectives:**

The aim of this study was to investigate the relationship between depression and sarcopenia risk in older adults with diabetes, with a particular focus on the potential mediating roles of nutrition and physical activity. The findings would provide empirical evidence to inform future prevention and intervention strategies.

**Design:**

A multi-center cross-sectional study.

**Methods:**

A total of 312 older adult patients with diabetes were selected from two hospitals using a convenience sampling method. The study evaluated demographic and clinical characteristics, along with factors such as nutrition, physical activity, depression, and sarcopenia risk. A multivariate logistic regression model was used to assess the association between depression and sarcopenia risk. Furthermore, the bootstrap resampling method, involving 5,000 samples and a 95% confidence interval (CI), was employed to examine the parallel mediating effects and determine whether nutrition and physical activity mediated the relationship between depression and sarcopenia risk. Data analysis was conducted using SPSS 26.0 and Mplus 7.0 software.

**Results:**

A total of 58.0% of participants were found to be at high risk for sarcopenia. Depression was shown to influence sarcopenia risk significantly, with nutrition and physical activity acting as parallel mediators. The total indirect effect was estimated at 0.087 (95% CI: 0.056, 0.126), accounting for 34.9% of the total effect (total effect = 0.249, 95% CI: 0.175, 0.311). Notably, nutrition played a crucial role in this mediation, contributing 61% of the total indirect effect.

**Conclusion:**

A significant proportion of older adults with diabetes were found to be at high risk of sarcopenia, which underscores the importance of routine screening for sarcopenia risk in older adults with diabetes. Depression was strongly associated with sarcopenia risk, with nutrition and physical activity acting as parallel mediators in this relationship. Given the pivotal role of nutrition in this parallel mediation model, the implementation of individualized nutrition plans is crucial for preventing muscle loss and reducing the risk of sarcopenia.

## 1 Introduction

Diabetes mellitus is one of the most prevalent chronic diseases globally and a leading cause of mortality. In 2019, the global number of older adults with diabetes was 135.6 million (19.3%), and this figure is projected to increase to 195.2 million by 2030 and 276.2 million by 2045 (1). China has the highest number of older adults diagnosed with diabetes among all countries (2). Older adults with diabetes typically face long-term disease management, a range of diabetes-related complications, substantial medical costs, and reduced life expectancy, all of which present significant public health challenges (3). Notably, depression and sarcopenia have become key areas of clinical and research focus due to their considerable impact on the overall health and quality of life of older adults with diabetes.

Sarcopenia, characterized by age-related declines in muscle strength, muscle mass, and/or physical performance, has increasingly been recognized as a global health concern (4). Studies have shown that diabetes increased the risk of sarcopenia by approximately 1.5-fold, with a reported prevalence as high as 38.3% among older adults with diabetes (5, 6). This elevated risk is largely attributed to factors such as chronic hyperglycemia, systemic inflammation, insulin resistance, diabetes-related complications, and adverse effects of antidiabetic medications (6). Sarcopenia is now considered a major complication of diabetes in older adults, and its severity tends to progress alongside the disease (3, 7). Evidence also suggested that older adult patients with diabetes and lower skeletal muscle mass (SMM) exhibit poorer glycemic control compared to those with higher SMM (8). Sarcopenia not only exacerbates diabetes-related health risks but also contributes to a vicious cycle of declining physical function, increased cardiovascular events, and higher mortality among older diabetic individuals (9). These effects severely impair quality of life in older adults with diabetes and place a growing burden on healthcare systems. Collectively, these factors complicate disease management and negatively affect quality of life. Given the substantial clinical implications, early identification and assessment of individuals at high risk for sarcopenia in this population are critical for enabling timely and effective prevention and intervention strategies. However, obtaining data using dual-energy X-ray absorptiometry (DXA) and bioelectrical impedance analysis (BIA) remains challenging due to the high cost of equipment, limited portability and accessibility, time-consuming procedures, and the requirement for specialized personnel. To address these limitations, the Asian Working Group for Sarcopenia (AWGS) 2019 recommended practical, validated, and cost-effective screening tools for sarcopenia, including the Strength, Assistance with Walking, Rise from a Chair, Climb Stairs, and Falls (SARC-F) questionnaire, the SARC-CalF, and the measurement of calf circumference (CC).

Depression, characterized by persistent sadness, loss of interest, and fatigue, involves both psychological and behavioral changes. Research indicated that approximately 33% of older adults with diabetes experienced depressive symptoms, often driven by concerns about complications, the burden of long-term self-care, and fears related to hypoglycemia and hyperglycemia (10). Depression not only impaired diabetes management but also contributed to poorer glycemic control and increased the risk of diabetes-related complications, which may in turn promote the development of sarcopenia (11). Several studies have investigated the relationship between depression and sarcopenia risk. A systematic review and meta-analysis reported that individuals with depression had a significantly higher likelihood of sarcopenia (odds ratio [OR] = 1.57, *p* < 0.001) (12). Similarly, Tsekoura et al. (13) found a positive correlation between depression and sarcopenia risk (*r* = 0.55, *p* < 0.001) (13).

Moreover, the relationship between depression and sarcopenia risk may be partially explained by shared contributing factors, such as inadequate nutritional intake and reduced physical activity (14). Individuals with depressive symptoms frequently exhibit poor nutritional status and low levels of physical activity, both of which are associated with declines in muscle strength and mass (15). Evidence suggested that malnutrition or risk of malnutrition was significantly associated with increased sarcopenia risk (16). Additionally, a cross-sectional study proposed that nutrition may mediate the relationship between depression and components of sarcopenia (17); however, the study did not include formal mediation analysis to confirm this hypothesis. Besides, depression-related physical inactivity may also contribute to sarcopenia. Prolonged inactivity may lead to structural and functional deterioration of muscle tissue, resulting in reduced muscle mass (18). Furthermore, sarcopenia severity has been shown to be negatively correlated with physical activity (*r* = −0.164, *p* = 0.006) (18). Taken together, these findings suggested that inadequate nutrition and low physical activity may mediate the relationship between depression and sarcopenia risk. Nevertheless, empirical evidence supporting this pathway remains limited, and the underlying mechanisms require further investigation.

**Figure 1:**
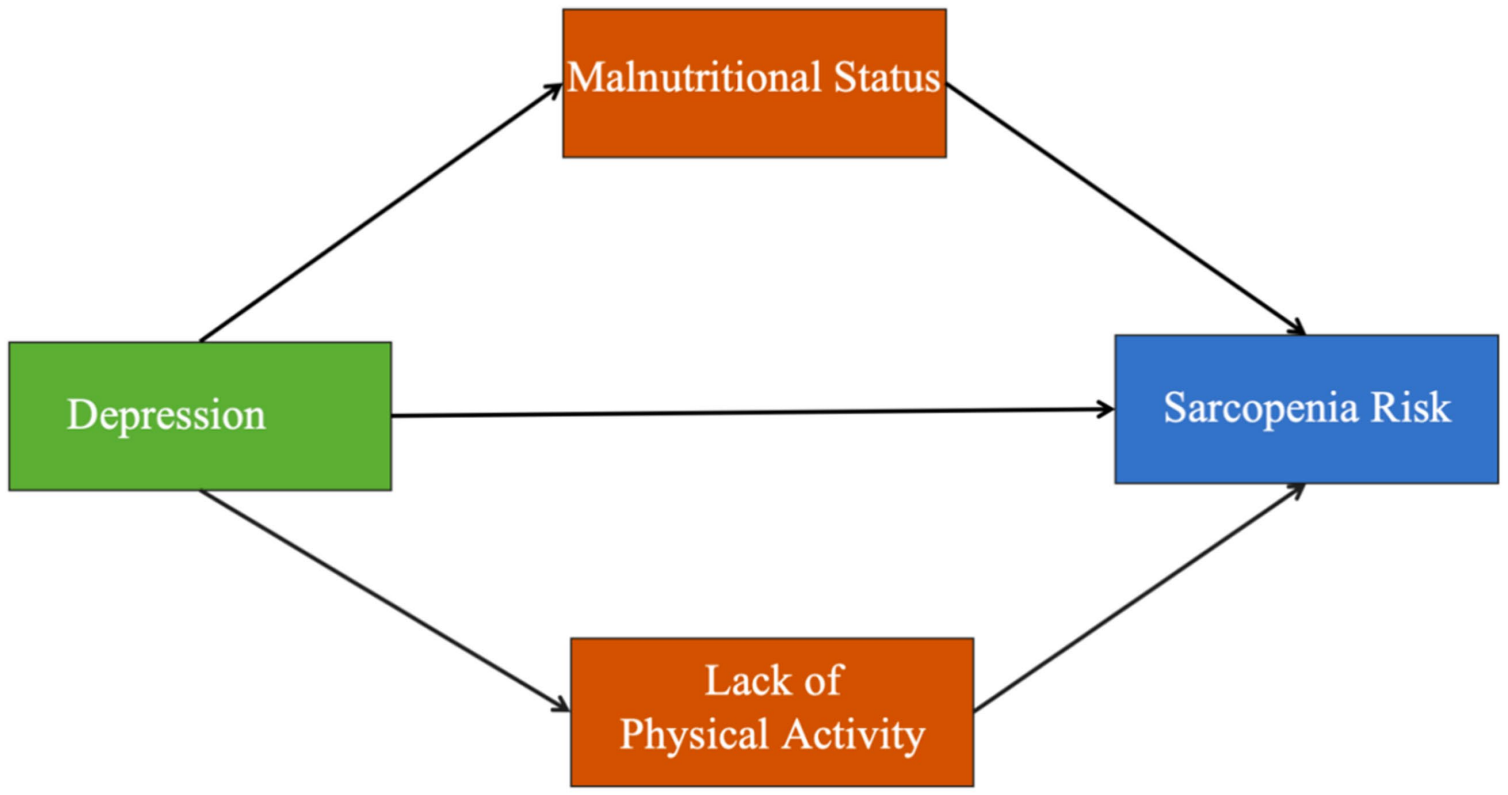
Hypothesized model of parallel mediation model.

In summary, research on the mechanisms and pathways between depression and sarcopenia risk remains limited among older adult diabetic patients. This study aimed to clarify the association between depression and sarcopenia risk and to examine whether nutrition and physical activity act as mediators in this relationship through a parallel mediation model. The hypothesized model is presented in Figure 1. The findings are expected to help clinicians better identify high-risk patients and deliver personalized treatment, improving health management and quality of life for older adults with diabetes.

## 2 Methods

### 2.1 Study design and participants

A multi-center cross-sectional study employing convenience sampling was conducted in Guangzhou, China from 2022 to 2023. The study adhered to the Strengthening the Reporting of Observational Studies in Epidemiology (STROBE) guidelines for reporting its findings (19).

The inclusion criteria for participants were: (1) age 60 years or older, (2) a confirmed diagnosis of diabetes, and (3) willingness to participate voluntarily. Exclusion criteria were: (1) presence of cognitive impairment, and (2) severe comorbid conditions, such as advanced heart, lung, or kidney disease.

### 2.2 Ethical considerations

Approval for this study was obtained from the Ethics Review Committee of the School of Nursing, Sun Yat-sen University (Number: L2022SYSU-HL-086). Prior to data collection, participants were fully informed of the study’s objectives and significance, and written informed consent was obtained. They were also assured that participation was voluntary and that they could withdraw from the study at any time during the questionnaire process.

### 2.3 Research instruments

#### 2.3.1 Sarcopenia risk

In this study, sarcopenia risk was assessed using calf circumference (CC) measurement, the SARC-F questionnaire, and the SARC-CalF tool, as recommended by the AWGS 2019. These tools were selected to capture multiple dimensions of sarcopenia risk and enhance assessment accuracy. For CC measurement, both calves were measured using a non-elastic measuring tape to determine the maximum circumference. And according to a recent study, the sensitivity and specificity of CC were 83.3 and 62.8%, respectively, based on the diagnostic criteria of the AWGS 2019 (20). Participants were considered at high risk for sarcopenia if the maximum circumference was <34 cm for males or <33 cm for females. The SARC-F questionnaire comprises five items—strength, assistance in walking, rising from a chair, climbing stairs, and falls—each scored from 0 (no difficulty) to 2 (very difficult or unable). Although the SARC-F has high specificity (90%), its sensitivity is relatively low (approximately 20%) (21). To improve diagnostic performance, the SARC-CalF tool combines the SARC-F score with CC measurement. This composite approach has been shown to increase the sensitivity and overall accuracy of sarcopenia risk screening, and has been identified in several studies as an optimal tool for older adults in China with a specificity of 94.6% and sensitivity of 60% (22). The SARC-CalF is scored by summing the SARC-F score and an additional score based on CC.

In this study, participants were classified as being at high risk for sarcopenia based on the AWGS 2019 screening criteria if they met any of the following conditions: (1) CC < 34 cm for males or <33 cm for females, (2) SARC-F score ≥4, or (3) SARC-CalF score ≥11.

#### 2.3.2 Depression

The 15-item Geriatric Depression Scale (GDS-15) was used to assess participants’ depressive symptoms over the past week, encompassing four dimensions: positive affect, negative affect, feelings of inferiority/disinterest, and uncertainty. Each item was scored dichotomously (0 or 1), yielding a total score ranging from 0 to 15, with higher scores indicating greater severity of depressive symptoms. A total score of ≥8 was considered indicative of possible depression in older adults. The GDS-15 demonstrated acceptable internal consistency in our diabetic cohort (Cronbach’s *α* = 0.662). While slightly lower than community samples [*α* = 0.745 in (23)]. This discrepancy may be attributed to the complex clinical profiles of diabetic patients, who frequently present with multimorbidity and heterogeneous symptom manifestations.

#### 2.3.3 Nutrition

Participants’ nutritional status was evaluated using the Mini-Nutritional Assessment–Short Form (MNA-SF), which comprises six items assessing weight loss, recent illness, body mass index (BMI), dietary intake over the past 3 months, psychological stress, and mobility. Each item is scored on a scale of 0–2 or 0–3, yielding a total score ranging from 0 to 14. A score of 8–11 indicates a risk of malnutrition, while a score of 0–7 indicates malnutrition. Related studies have demonstrated that the MNA-SF exhibited moderate sensitivity (59.2%) and relatively high specificity (78.8%) in diagnosing malnutrition among older adults, and the area under the receiver operating characteristic curve (AUC) was 0.77 (24).

#### 2.3.4 Physical activity

The International Physical Activity Questionnaire (Short Form) (IPAQ-SF) was used to assess the participants’ physical activity levels, included 5 components: household chores and gardening, leisure activities, work-related physical activities, transportation-related physical activities, sedentary behavior. The IPAQ-SF collected related information from the past week and calculated total energy expenditure. Based on the standards of the International Physical Activity Working Group, participants’ physical activity levels were categorized into high, moderate and low. Studies have consistently shown that the IPAQ-SF demonstrated acceptable reliability in Chinese populations, with an intraclass correlation coefficient (ICC) of 0.79 (25).

#### 2.3.5 Potential covariates

Based on previous research, several potential covariates were collected through face-to-face interviews conducted by trained researchers. These included age, gender, place of residence, educational level, marital status, type of medical insurance, duration of diabetes, diabetes-related complications, family history of diabetes, polypharmacy, smoking and alcohol use, BMI, and glycated hemoglobin (HbA1c). BMI was calculated as weight in kilograms divided by height in meters squared (kg/m^2^), and HbA1c values were obtained from the most recent laboratory results recorded by the researchers.

### 2.4 Data analysis

Data analysis was conducted using SPSS 26.0 and Mplus 7.0. Categorical variables were expressed as frequencies and percentages, while continuous variables were presented as means ± standard deviations (SD) or medians, as appropriate. The chi-square test was used to assess differences in the distribution of sarcopenia risk. A multivariate logistic regression model with a hierarchical block design was employed to evaluate the independent contributions of sociodemographic characteristics, medical conditions, and patient-centered variables to sarcopenia risk. Parallel mediation analysis was performed using Mplus 7.0 to examine the relationships among depression (independent variable), malnutrition status and physical inactivity (mediators), and sarcopenia risk (dependent variable). The mediators and outcome variables were treated as categorical: malnutritional status (0 = normal, 1 = at risk, 2 = malnourished), physical activity level (0 = high, 1 = moderate, 2 = low), and sarcopenia risk (0 = low, 1 = high). This study used weighted least squares mean and variance adjusted estimation (WLSMV) combined with threshold parameter method to estimate the parameters of a structural equation model (SEM) containing categorical mediator variables, and evaluated the significance and 95% confidence interval (CI) of the mediation effect through bias corrected Bootstrap program (5,000 resampling). An indirect effect was considered statistically significant if the 95% CI did not include zero. Model fit was evaluated using the following indices: Root Mean Square Error of Approximation (RMSEA) (<0.05 for excellent fit, <0.08 for acceptable), Comparative Fit Index (CFI) (>0.90 for excellent, >0.80 for acceptable), Tucker–Lewis Index (TLI) (>0.90 for excellent, >0.80 for acceptable), and Standardized Root Mean Square Residual (SRMR) (<0.05 for excellent fit, ≦0.08 for acceptable) (26). A two-sided *p*-value <0.05 was considered statistically significant.

## 3 Results

### 3.1 Characteristics of participants

The characteristics of the participants were presented in Table 1. A total of 312 older adults with diabetes were enrolled, with an average age of (68.79 ± 6.73) years. A total of 181 participants (58.0%) were identified as having high sarcopenia risk (Table 2).

**Table 1:** Characteristics of participants (*N* = 312).

Variables	*N*	Low sarcopenia risk	High sarcopenia risk	*χ^2^*	*P*
		*n* (%)			
Gender				0.051	0.822
Male	162	69 (42.6)	93 (57.4)		
Female	150	62 (41.3)	88 (58.7)		
Age (years)				9.982	0.041
60 ~ 65	99	47 (47.5)	52 (52.5)		
65 ~ 70	88	42 (47.7)	46 (52.3)		
70 ~ 75	62	24 (38.7)	38 (61.3)		
75 ~ 80	42	15 (35.7)	27 (64.3)		
≥80	21	3 (14.3)	18 (85.7)		
Habitation				0.123	0.726
City	269	114 (42.4)	155 (57.6)		
Countryside	43	17 (39.5)	26 (60.5)		
Marital status				0.055	0.814
Married	265	112 (42.3)	153 (57.7)		
Unmarried, divorce	47	19 (40.4)	28 (59.6)		
Education level				4.155	0.385
Primary school and below	67	27 (40.3)	40 (59.7)		
Middle school	96	40 (41.7)	56 (58.3)		
High school	78	29 (37.2)	49 (62.8)		
Junior College	27	16 (59.3)	11 (40.7)		
Bachelor degree or above	44	19 (43.2)	25 (56.8)		
Medical payment				4.283	0.038
Medical insurance	244	95 (38.9)	149 (61.1)		
Self-funded	68	36 (52.9)	32 (47.1)		
Duration of diabetes (years)				5.986	0.200
<5	92	47 (51.1)	45 (48.9)		
5 ~ 10	45	16 (35.6)	29 (64.4)		
10 ~ 15	80	34 (42.5)	46 (57.5)		
15 ~ 20	27	8 (29.6)	19 (70.4)		
≥20	68	26 (38.2)	42 (61.8)		
Diabetes-related complications				2.136	0.144
Yes	198	77 (38.9)	121 (61.1)		
No	114	54 (47.4)	60 (52.6)		
Family history				0.010	0.922
Yes	132	55 (41.7)	77 (58.3)		
No	180	76 (42.2)	104 (57.8)		
Polypharmacy				0.632	0.427
Yes	180	79 (43.9)	101 (56.1)		
No	132	52 (39.4)	80 (60.6)		
Smoking status				5.941	0.051
Never	216	87 (40.3)	129 (59.7)		
Quit smoking	47	27 (57.4)	20 (42.6)		
Smoking	49	17 (34.7)	32 (65.3)		
Drinking status				0.188	0.910
Never	216	91 (42.1)	125 (57.9)		
Quit smoking	60	24 (40.0)	36 (60.0)		
Smoking	36	16 (44.4)	20 (55.6)		
BMI (Kg/m^2^)				39.342	<0.001
<18.5^①^	11	1 (9.1)	10 (90.9)		①③*
18.5 ~ 24.0^②^	178	55 (30.9)	123 (69.1)		②③*
24.0 ~ 28.0^③^	94	62 (66.0)	32 (34.0)		
≥28.0^④^	29	14 (48.3)	15 (51.7)		
HbA1c (%)				0.209	0.647
<7.5%	137	56 (40.9)	81 (59.1)		
≥7.5%	175	75 (42.9)	100 (57.1)		
Nutritional status				25.662	<0.001
Normal	203	106 (52.2)	97 (47.8)		
Malnutrition risk	100	26 (26.0)	74 (74.0)		
Malnutrition	9	0 (0.0)	9 (100.0)		
Physical activity				5.841	0.054
Low level	31	8 (25.8)	23 (74.2)		
Moderate level	128	50 (39.1)	78 (52.3)		
High level	153	73 (47.7)	80 (52.3)		
Depression status				8.361	0.004
Yes	11	0 (0.0)	11 (100.0)		
No	301	132 (43.9)	169 (56.1)		

Pairwise comparison between groups was developed by using Bonferroni method, *p < 0.05. ^①②③④^Number used for Pairwise comparison between groups.

**Table 2:** Screening results of sarcopenia risk.

Categorization	SARC-F	CC	SARCF-CalF	Total
	*n* (%)			
High sarcopenia risk	57 (18.3%)	139 (44.6%)	101 (32.4%)	181 (58.0%)
Low sarcopenia risk	255 (81.7%)	173 (55.4%)	211 (67.6%)	131 (42.0%)

### 3.2 Associations between depression and sarcopenia risk

To examine the association between depression and sarcopenia risk, three models were formulated (Table 3). Consistent findings indicated a positive relationship between depression and sarcopenia risk [Model 3: OR = 1.288, 95%CI: (1.215, 1.366), *p* < 0.001]. Besides, multicollinearity was assessed via variance inflation factors (VIF < 5 acceptable) and model 3 showed no multicollinearity diagnostics (VIF: 1.019–1.628). To evaluate models hierarchy, nested likelihood-ratio tests were conducted to compare sequentially specified models. These tests revealed statistically significant hierarchical improvements across all model comparisons (all *p* < 0.001).

**Table 3:** Associations between depression and sarcopenia risk.

Covariates	Model 1		Model 2		Model 3	
	OR (95%CI)	*P*	OR (95%CI)	*P*	OR (95%CI)	*P*
Depression scores	1.198 (1.145, 1.254)	<0.001	1.204 (1.147, 1.264)	<0.001	1.288 (1.215, 1.366)	<0.001
Age	—	—	1.060 (1.043, 1.077)	<0.001	1.058 (1.037, 1.078)	<0.001
Gender: Female (ref)						
Male	—	—	0.985 (0.802, 1.208)	0.881	0.997 (0.754, 1.139)	0.986
Habitation: City (ref)						
Countryside	—	—	0.918 (0.689, 1.222)	0.556	0.862 (0.623, 1.194)	0.373
Marital status: Married (ref)						
Unmarried, divorce	—	—	0.800 (0.602, 1.064)	0.125	0.727 (0.530, 0.999)	0.050
Education level: Primary school and below (ref)						
Middle school	—	—	1.161 (0.882, 1.530)	0.287	1.851 (1.341, 2.555)	<0.001
High school	—	—	1.383 (1.034, 1.851)	0.029	2.281 (1.631, 3.190)	<0.001
Junior College	—	—	0.451 (0.304, 0.670)	<0.001	0.628 (0.403, 0.976)	0.039
Bachelor degree or above	—	—	0.909 (0.639, 1.294)	0.597	1.681 (1.109, 2.549)	0.014
Medical payment: Medical insurance (ref)						
Self-funded	—	—	0.478 (0.376, 0.607)	<0.001	0.574 (0.434, 0.758)	<0.001
Duration of diabetes (years)		—	—	—	1.019 (1.005, 1.034)	0.010
Complication: No (ref)						
Yes	—	—	—	—	0.920 (0.718, 1.177)	0.506
Family history: No (ref)						
Yes	—	—	—	—	0.835 (0.660, 1.057)	0.133
Polypharmacy: No (ref)						
Yes	—	—	—	—	0.758 (0.598, 0.961)	0.022
Smoking status: Never (ref)						
Quit smoking	—	—	—	—	0.290 (0.195, 0.430)	<0.001
Smoking	—	—	—	—	1.167 (0.809, 1.684)	0.408
Drinking status: Never (ref)						
Quit drinking	—	—	—	—	2.055 (1.433, 2.946)	<0.001
Drinking	—	—	—	—	1.115 (0.746, 1.665)	0.596
BMI (Kg/m^2^): 18.5 ~ 24.0 (ref)						
<18.5	—	—	—	—	2.667 (1.042, 6.828)	0.041
24.0 ~ 28.0	—	—	—	—	0.174 (0.135, 0.224)	<0.001
≥28.0	—	—	—	—	0.352 (0.244, 0.507)	<0.001
HbA1c (%): < 7.5 (ref)					0.917 (0.731, 1.150)	0.453
≥7.5	—	—	—	—		

OR, odds ratios; CI, confidence interval. Model 1: Crude model. Model 2: Adjusted for age, gender, habitation, educational level, marital status and medical payment. Model 3: Model 2+ duration of diabetes, diabetes-related complications, family history, polypharmacy, drinking status, smoking status, BMI, glycemic control (HbA1c).

### 3.3 Parallel mediation model

**Figure 2:**
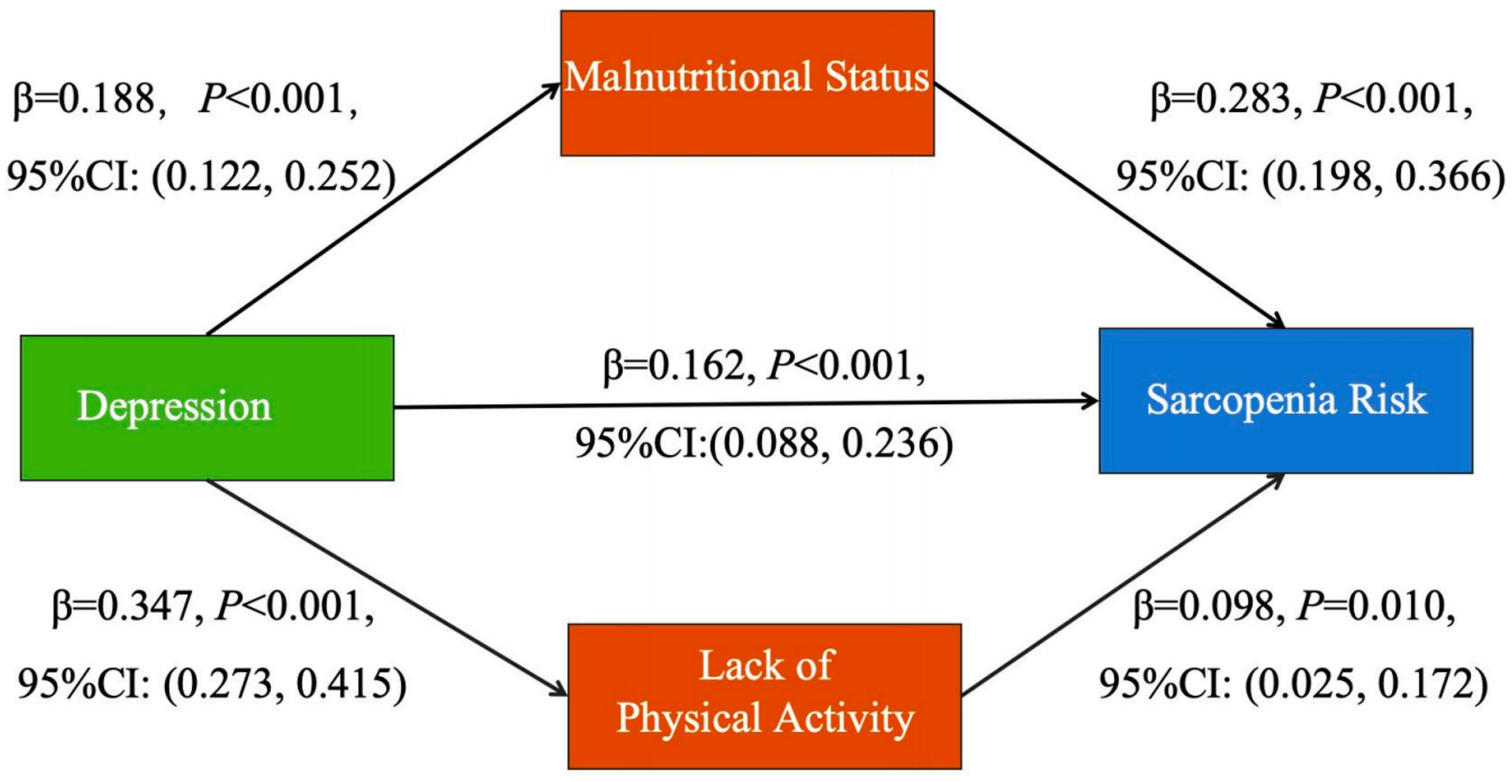
Parallel mediation model of depression, nutrition, physical activity and sarcopenia risk. Age, medical payment and BMI were set as control variables for the model.

Detailed model specifications were presented in Figure 2. After adjusting for covariates (age, medical payment, BMI) on sarcopenia risk (all *p* < 0.05 in Table 1), results revealed that the parallel mediation model demonstrated good fit indices: RMSEA = 0.020, CFI = 0.996, TLI = 0.988 and SRMR = 0.078. The total indirect effect of the parallel mediation model was calculated as 0.087 (95% CI: 0.056, 0.126), explaining 34.9% of the total effect (total effect = 0.249, 95% CI: 0.175, 0.311). Notably, malnutritional status (*β* = 0.053, 95%CI: 0.032, 0.087) played a pivotal role in this mediation, contributing 61% of the total indirect effect, while lack of physical activities (β = 0.034, 95%CI: 0.009, 0.063) accounted for the remaining 39%.

### 3.4 Statistical power analysis

Post-hoc power for the multivariate logistic regression (Model 3) was assessed using G*Power 3.1. Based on the observed odds ratio for depression (OR = 1.288), sarcopenia risk prevalence of 58.0% and sample size of 312, the analysis yielded power of 99.98% (*α* = 0.05).

## 4 Discussion

A total of 312 participants were enrolled in the study, with 58.0% identified as being at high risk for sarcopenia. Multivariate logistic regression analysis indicated that depression was significantly associated with an increased risk of sarcopenia, even after controlling for potential confounding variables. Moreover, parallel mediation analysis revealed that both nutritional status and physical activity levels mediated the relationship between depression and sarcopenia risk among older adults with diabetes.

In our study, 58.0% of older adults with diabetes were identified as being at high risk for sarcopenia, a rate significantly higher than those reported in previous studies. Ida et al. (21) found a 20.7% risk using the SARC-F tool (11), while Massimino et al. (27) reported a rate of 8.7% (27). These discrepancies were likely due to differences in diagnostic tools and participants characteristics. Both of the aforementioned studies used the SARC-F tool, which was known for its limited sensitivity (ranging from 18.2 to 33.3%), potentially leading to screening bias (28). In contrast, our study employed consensus-recommended parallel tools to improve screening accuracy. Additionally, our participants exhibited a high prevalence of poor glycemic control (56.1%) and diabetes-related complications (63.5%), factors that have been identified as contributing to an increased risk of sarcopenia. Despite the acknowledged importance of early detection and intervention, awareness of sarcopenia remains low. Only 9% of community-dwelling adults and 13–21% of healthcare professionals were familiar with its diagnosis (29, 30). Therefore, enhancing educational efforts for both healthcare providers and the general public is crucial. By emphasizing the risks and available mitigation strategies, we can improve self-care motivation and ultimately reduce the risk of sarcopenia.

Depression was significantly associated with an increased risk of sarcopenia. Previous studies have demonstrated that individuals with depression were at higher risk for sarcopenia, particularly in older adults with diabetes (21). A study involving 447 participants found that depressive symptoms were independently linked to decreased muscle mass (*r* = −0.382) and muscle strength (*r* = −0.228) (31). Additionally, a Mendelian randomization study revealed that depression was associated with lower hand grip strength, a key indicator of sarcopenia (32). Cox proportional hazards regression analysis further confirmed that depression was significantly associated with an increased risk of developing sarcopenia (hazard ratio = 1.34; 95% CI: 1.19, 1.50) over a mean follow-up period of 3.53 years (33). Depression often induced elevated inflammation, hormonal imbalances, and neurotransmitter dysfunction, all of which contribute to muscle mass deterioration (12). In older adult diabetic patients, depression can lead to apathy, impairing their ability to manage health, worsening hyperglycemia, and disrupting insulin metabolism. Notably, depression has been identified as a key predictor of poor glycemic control, which was a significant risk factor for sarcopenia (34). The association between depression and sarcopenia risk underscores the complex interplay between mental health, glycemic control, and muscle deterioration. Early identification of depression enables timely interventions that can prevent or delay sarcopenia. Therefore, personalized care plans to support older adult patients with depression are essential for mitigating the risk of sarcopenia. Given the chronic nature of depression and sarcopenia, long-term follow-up and regular reassessment are essential. Tailoring interventions to address the patient’s evolving needs and progress—incorporating periodic evaluations of muscle mass, strength, and mental health—can help sustain treatment efficacy and further mitigate the risk of sarcopenia over time.

It was important to note that nutrition played a key role in mediating the relationship between depression and sarcopenia risk, accounting for 61% of the total indirect effect. This suggested that the parallel mediation effects were dominantly mediated through nutrition among older adult patients with diabetes. In traditional Chinese culture, eating was not only a biological necessity but also an important social activity as meals were often shared with family and friends. However, individuals with depression frequently avoid social interactions. This social withdrawal could significantly impact their eating habits and they may skip meals or eat alone, further exacerbating the risk of malnutrition (35). Moreover, many older adult Chinese individuals adhere to dietary patterns that are primarily carbohydrate-heavy, often leading to a relative decrease in protein intake (16), which may aggravate the nutritional deficiency of older adult patients with diabetes. Unfortunately, depression further exacerbates this nutritional deficiency by impairing appetite, which reduces protein consumption. This reduction in protein intake would hinder the body’s ability to obtain essential nutrients necessary for muscle protein synthesis (15, 27). Therefore, nutritional interventions are crucial in counteracting the effects of depression-induced malnutrition, helping to ensure that older adult individuals receive the necessary nutrients to support muscle health and overall well-being. A meta-analysis has shown that continuous nutritional interventions, including protein supplementation, effectively improve muscle mass in older adults (36). However, this remains challenging for older adult diabetic patients. Aging is often accompanied by reduced appetite and impaired chewing and swallowing functions, leading to insufficient protein intake. Glycemic control further limits dietary options, with reduced consumption of common protein sources such as dairy and red meat, decreasing both the quantity and diversity of protein intake. High costs of protein foods and limited nutritional knowledge, coupled with cognitive decline, further hinder adequate dietary management. To address these issues, comprehensive, individualized interventions are essential. Modifying the diet structure by reducing carbohydrates and increasing protein is recommended as it is more sustainable and cost-effective. Nutrition education through community programs and regular healthcare visits can raise awareness of protein needs among patients and caregivers. Dietary plans should be tailored based on glycemic control, renal function, and oral health. These strategies are crucial for optimizing protein intake, preserving muscle mass, and reducing the risk of sarcopenia and related complications in older adult diabetic populations.

Physical activity also served as an important mediator between depression and sarcopenia risk with indirect effect of 0.034, contributing 39% of the total indirect effect. Maintaining physical activity was essential for older adult diabetic patients to stabilize blood glucose, enhancing muscle function, and preserving overall health. In a depressed state, patients often reduced their activity due to factors such as lack of motivation, fatigue, and feelings of isolation, which resulted in a notable decline in daily exercise (37). Low activity levels usually lead to diminished muscle use and stimulation, resulting in slowed muscle protein synthesis and a subsequent loss of muscle mass (38). Additionally, insufficient physical activity may reduce insulin sensitivity, worsen metabolic disturbances, and accelerated the decline in muscle strength and endurance, which further contribute to the development of sarcopenia (39). Thus, the restriction of physical activity due to depression accelerates the cumulative risk of sarcopenia in older adult diabetic patients. Exercise has been well-documented for its positive effects on both mental and physical health (40), particularly in enhancing blood sugar control and muscle quality. Exercise induces the release of myokines, which have anti-inflammatory effects, playing a crucial role in reducing systemic inflammation. Chronic inflammation, a common feature of both depression and diabetes, contributes to insulin resistance and poor glycemic control. By modulating inflammatory markers such as CRP, IL-6, and TNF-α, exercise can help alleviate both the physical and psychological burdens of diabetes (40). This anti-inflammatory action not only improves muscle health further to prevent sarcopenia but also enhances glycemic control, reducing the risk of diabetes-related complications. Particularly, resistance training has been highlighted as a key intervention for preventing sarcopenia (7). However, despite its numerous benefits, designing suitable and effective exercise programs for older adult diabetic patients remains a challenge, especially when considering the additional burden of depression. The impact of exercise on muscle mass is highly time-dependent and influenced by factors such as frequency, duration, intensity, and volume (41). Therefore, developing personalized exercise strategies that account for these variables and the unique needs of older adult diabetic patients is essential. Given that depression contributes to physical inactivity and exacerbates the risk of sarcopenia, integrating mental health support into exercise programs is crucial. Providing emotional support and encouraging social exercise activities can improve adherence to exercise plans (42). This dual approach not only reduces depressive symptoms but also enhances the effectiveness of exercise, ultimately leading to improved muscle health, better glycemic control, and an enhanced quality of life for older adult patients.

## 5 Limitations

This study has several limitations that should be acknowledged. First, nutrition and physical activity were assessed primarily through self-reported measures, without the use of objective assessment tools. Although self-reporting is commonly used in research, it is subject to recall bias, which may affect the accuracy of participants’ reports regarding their dietary intake and physical activity levels, potentially influencing the study’s findings. Second, the effects of nutrition and physical activity on muscle mass are complex, time-dependent, and influenced by multiple factors. However, the cross-sectional design of this study limits the ability to infer causal relationships among sarcopenia risk, depression, nutrition, and physical activity. Future studies using longitudinal designs are needed to better elucidate the temporal and causal relationships among these variables. Third, the study sample consisted exclusively of older adults with diabetes, which may limit the generalizability of the findings to other populations. Future research should include more diverse cohorts to determine whether the observed associations among depression, nutrition, physical activity, and sarcopenia risk hold across different demographic and clinical populations. Fourth, this study used convenience sampling method from two tertiary hospitals in Guangzhou, which may cause the insufficient representation of community residents and remote patients. Future studies are suggested to expand the sampling range, in order to enhance external validity. In addition, this study overlooked the record of participants who refused. In future studies, a pre-screening log system is recommended to be established to fully record the sampling and survey process and increase research visibility.

## 6 Relevance for clinical practice

The findings of this study offer several important implications for clinical practice. First, the high prevalence of sarcopenia risk among older adults with diabetes highlights the urgent need for early screening, prevention, and targeted intervention strategies. It is essential that both healthcare providers and patients are well-informed about the definitions, causes, and management of sarcopenia. Educational initiatives can help raise sarcopenia awareness and empower patients to make informed decisions about their nutritional intake and physical activity, which was significantly associated with sarcopenia risk. Second, given the strong association between depression and sarcopenia risk in this population, routine screening for both conditions should be integrated into clinical practice. Early identification of depressive symptoms and sarcopenia can facilitate timely, appropriate interventions to reduce related health risks. Third, depression may increase sarcopenia risk indirectly through its negative impact on nutritional status and physical activity. Considering the complex health needs of older adults with diabetes, a multidisciplinary approach is essential. Collaborative care involving endocrinologists, geriatricians, dietitians, mental health professionals, and nurses can ensure the development of comprehensive, individualized care plans that address both physical and mental health. Finally, given the pivotal role of nutrition in mediating the relationship between depression and sarcopenia risk, healthcare professionals should prioritize the development of personalized nutrition plans. These plans should aim not only to correct nutritional deficiencies but also to optimize dietary intake in a way that supports mental well-being and helps prevent muscle loss.

## 7 Conclusion

Older adults with diabetes demonstrated a high prevalence of sarcopenia risk, which was significantly associated with depression. Notably, this study identified nutrition and physical activity as key mediating factors in the relationship between depression and sarcopenia risk. These findings underscore the importance of prioritizing sarcopenia risk assessment and management in this population. Clinical practitioners and community health professionals should implement targeted strategies for early identification, prevention, and intervention to prevent the development of sarcopenia. Furthermore, there is a critical need for qualitative and longitudinal research, as well as population-based surveys assessing sarcopenia awareness to deepen the understanding of these complex interrelationships and to generate robust, systematic evidence.

## Data Availability

The original contributions presented in the study are included in the article/supplementary material, further inquiries can be directed to the corresponding author.
